# Annexin A2 could enhance multidrug resistance by regulating NF-κB signaling pathway in pediatric neuroblastoma

**DOI:** 10.1186/s13046-017-0581-6

**Published:** 2017-08-16

**Authors:** Yi Wang, Kai Chen, Yihong Cai, Yuanxia Cai, Xiaojun Yuan, Lifeng Wang, Zhixiang Wu, Yeming Wu

**Affiliations:** 1 Department of Pediatric Surgery, Xinhua Hospital Affiliated to Shanghai Jiao Tong University School of Medicine, No.1665, Kongjiang Road, Yangpu District, Shanghai, China; 2 Pediatric Hematology & Oncology Department, Xinhua Hospital Affiliated to Shanghai Jiao Tong University School of Medicine, No.1665, Kongjiang Road, Yangpu District, Shanghai, China; 3 Pathology Department, Xinhua Hospital Affiliated to Shanghai Jiao Tong University School of Medicine, No.1665, Kongjiang Road, Yangpu District, Shanghai, China; 4 Division of Pediatric Oncology, Shanghai Institute of Pediatric Research, No. 1665, Kongjiang Road, Yangpu District, Shanghai, China

**Keywords:** Proteomics, ANXA2, Chemotherapy, Drug resistance, NF-κB, Neuroblastoma

## Abstract

**Background:**

Chemotherapy is one of major therapeutic regimens for neuroblastoma (NB) in children. However, recurrence and metastasis associated with poor prognosis caused by acquired multidrug resistance remains a challenge. There is a great need to achieve new insight into the molecular mechanism of drug resistance in NB. The aim of this study is to identify novel drug sensitivity-related biomarkers as well as new therapeutic targets to overcome chemoresistance.

**Methods:**

We proteome-wide quantitatively compared protein expression of two NB cell lines with different drug sensitivities, isolated from the same patient prior to and following chemotherapy. Annexin A2 (ANXA2) emerged as a key factor contributing to drug resistance in NB. Then, we assessed the correlation of ANXA2 expression and clinical characteristics using a tissue microarray. Further, the roles of ANXA2 in chemoresistance for NB and the underlying mechanisms were studied by using short hairpin RNA (shRNA) in vitro and vivo.

**Results:**

First in total, over 6000 proteins were identified, and there were about 460 significantly regulated proteins which were up- or down-regulated by greater than two folds. We screened out ANXA2 which was upregulated by more than 12-fold in the chemoresistant NB cell line, and it might be involved in the drug resistance of NB. Then, using a tissue chip containing 42 clinical NB samples, we found that strong expression of ANXA2 was closely associated with advanced stage, greater number of chemotherapy cycles, tumor metastasis and poor prognosis. Following knockdown of ANXA2 in NB cell line SK-N-BE(2) using shRNA, we demonstrate enhanced drug sensitivity for doxorubicin (2.77-fold) and etoposide (7.87-fold) compared with control. Pro-apoptotic genes such as AIF and cleaved-PARP were upregulated. Inhibiting ANXA2 expression attenuated transcriptional activity of NF-κB via down-regulated nuclear translocation of subunit p50. Finally, simulated chemotherapy in a xenograft NB nude mouse model suggests that ANXA2 knockdown could improve clinical results in vivo.

**Conclusion:**

Our profiling data provided a rich source for further study of the molecular mechanisms of acquired drug resistance in NB. Further study may determine the role of ANXA2 as a prognostic biomarker and a potential therapeutic target for patients with multidrug-resistant NB.

**Electronic supplementary material:**

The online version of this article (doi:10.1186/s13046-017-0581-6) contains supplementary material, which is available to authorized users.

## Background

Neuroblastoma (NB) is the most common pediatric extra-cranial tumor, derived from precursor cells of the sympathetic nervous system. This malignant tumor is responsible for approximately 10–15% of childhood cancer deaths [1, 2]. Chemotherapy is the principal treatment, especially for patients in advanced stages of the disease. Despite advances in therapy over time, 5-year event-free survival in children with high risk NB is less than 31% [3, 4].

Treatment effectiveness is limited by acquired multidrug resistance. In recent years, several efforts have been undertaken to identify genes and proteins associated with drug resistance. Studies have suggested that some high-level drug resistance observed in recurrent NB is attributable to p53 mutations and/or loss-of-function [5]. Overexpression of multidrug resistance protein 1 (MRP1) correlates with chemoresistance in several biological processes, including tumor relapse and metastasis [6, 7].

With the recent development of mass spectrometry-based proteomics, additional genes have been associated with multidrug resistance [8, 9]. This technology permits analysis of the modification status of proteins at a macroscopic level, and provides a platform for screening key protein and/or biomarkers in cancer research [10].

In this study, we quantitatively analyzed the proteome changing in two NB cell lines, SK-N-BE (1) and SK-N-BE (2), both of which were isolated from the same patient. Each line was demonstrated different drug sensitivities. We focused on AnnexinA2 (ANXA2) following bioinformatic analysis and literature review. We determined that ANXA2 expression correlated with greater number of chemotherapy cycles and poor prognosis. We propose that ANXA2 may play a central role in chemoresistance. We show that knocking down ANXA2 may enhance drug sensitivity of NB cells both in vitro and in vivo. In addition, inhibiting ANXA2 expression may downregulate transcriptional activity of NF-κB. Our results provide new insights into the role of ANXA2 in NB chemoresistance.

## Methods

### Cell lines and cell culture

The human neuroblastoma cell lines SK-N-BE(1) and SK-N-BE(2) were obtained from Children’s Oncology Group (COG, USA). SK-N-BE(1) cell line was isolated from bone marrow of a 2-year-old patient when he was primary diagnosed without any treatment, and SK-N-BE(2) cell line was gained from the same patient when he has been received several courses of chemotherapy with cyclophosphamide, doxorubicin, as well as vincristine. These two cell lines had different drug sensitivity (Additional file 1: Figure S1) [11–13].

SK-N-BE (1) and SK-N-BE (2) cell lines were maintained in RPMI 1640 (Gibco, USA) with 10% fetal bovine serum (Gibco, USA), 50 units/ml penicillin, 50 μg/ml streptomycin (Yeasen, China) and 1X ITS (Sodium Pyruvate 0.11 g/L, L-glutamine 1.5 g/L, NaHCO3 1.5 g/L (Yeasen, China)). These two cell lines were incubated humidified air supplemented 5% CO2 at 37 °C.

To enable the quantitative proteomics, we used specialized stable isotope labeling with amino acids in cell culture (SILAC) medium supplemented with 10% dialyzed fetal bovine serum (Invitrogen, Darmstadt, Germany) to label the cells, where deficient Arginine and Lysine were supplemented with either stable isotope encoded heavy Arginine and Lysine (Euriso-top) or normal Arginine and Lysine for the light. Labeling efficiency was confirmed before the following quantitative full proteome analysis.

### Protein extraction and in-solution digestion and peptides pre-separation

After washing with pre-cold phosphate-buffered saline (PBS, Mediatech Inc., Manassas, VA), equal number of SILAC coded SK-N-BE (1) and SK-N-BE (2) cells were mixed and lyzed with chilled lysis buffer, 8 M urea in 100 mM NH_4_HCO_3_ with 1× protease inhibitor cocktail (Roche Diagnostic GmbH, Germany) and incubated on ice for half an hour. Cell debris were removed by centrifugation and the supernatant was collected. Protein concentration in lysates was determined by the Bradford assay accordingly. The extracted proteins were digested by trypsin according to the protocol. Briefly, after the alkylation and reduction, protein lysate was diluted four times with 100 mM NH_4_HCO_3_ (pH 8.0) to decrease the urea concentration to 2 M. Trypsin was then added at an enzyme-substrate ratio of 1:100 (*w*/w). The digestion was subsequently performed at 37 °C for 16 h.

### High performance liquid chromatography (HPLC)/mass spectra (MS)/MS analysis

Tryptic digest was then pre-separated into 20 fractions with a reverse phase C18 Xbridge column (Waters Corp., Milford, MA) by a preparative HPLC system (Shimadzu, Japan) at high pH condition. Each fraction was lyophilized and further analyzed in a nano-HPLC coupled Orbitrap Q-Exactive mass spectrometer (Thermo Fisher Scientific, Waltham, MA). The raw spectra data was processed by Maxquant software (v1.4.1.2) [14]. MS/MS spectra data was searched against the Uniprot human database (88,817 sequences) by Andromeda search engine [15].

### Patients and tissue specimens

In our study, there were 42 pediatric patients with neuroblastoma (international neuroblastoma staging system (INSS) stage I–IV & IVs) who were histologically diagnosed in Xinhua hospital affiliated to Shanghai Jiaotong University School of Medicine during January 2006 and December 2011. All of the patients enrolled in the study had received chemotherapy before surgery or not, and all of them underwent surgery or biopsy. Each tumor specimen was stored in liquid nitrogen for tissue microarray analysis. The experimental protocols were approved by the Ethics Committee of the Xinhua hospital affiliated to Shanghai Jiaotong University School of Medicine. We analyzed the recorded data of each patient and performed follow-up through phone calls.

### Tissue microarray (TMA) preparation and Immunohistochemistry (IHC)

These tissue specimens were separated out a small part and shaped in the special mold for microarray preparation. After fixed in the 4% paraformaldehyde over night, they were trimmed and embedded in paraffin as a planned array. Then, samples were sectioned (5 μm) and attached to poly-L-lysine coated slides. Immunohistochemical staining was performed using a standard immunoperoxidase staining procedure (primary antibody, ANXA2, 1:100, CST, USA). Hematoxylin was used as a counterstain. The tissue sections were viewed independently by two pathologists in a blind fashion. IHC staining was graded on a specialized scale from 0 to 4: where 0 represented negative expression, 1 represented weakly positive expression (0–10% positive cells), 2 represented mildly positive expression (10–30% positive cells), 3 represented moderately positive expression (30–50% positive cells), 4 represented strongly positive expression (50–100% positive cells). The scale was determined according to the average number of positive cells in 10 random fields of one slide.

### Lentivirus-mediated interference for ANX A2

The pGMLV-SC5 RNAi and pGMLV-SB3 RNAi vector (GeneChem, Shanghai, China, Additional file 2: Figure S2) was used to package the lentivirus for knocking down the ANXA2 expression. The shRNA sequences were designed by selecting a specific target sequence for the human ANXA2 gene, as described by GeneChem (Additional file 3: Figure S3). These oligonucleotides were annealed and subcloned according to the manufacturer’s recommendations, and a non-targeting control shRNA (scrambled control) was obtained from GeneChem. Lentivirus was packaged according to the manufacturer’s protocol of GeneChem. The produced lentiviral particles named pGC-shANXA2-LV and pGC-vector-LV. Cells were infected with lentiviruses in the presence of polybrene (5 μg/ml) for 12 h, 37 °C incubation, and then the lentiviral medium was replaced by the fresh culture solution.

### Drug treatment and cell viability assay

Doxorubicin (Sigma, St. Louis, MO, USA) and Etoposide (Sigma) stock solution (10 mM) was prepared by dissolving it in DMSO, and solution was diluted as ten concentration gradients from 0 μM to 2 μM for doxorubicin and 0 μM to 16 μM for Etoposide before experiment. SK-N-BE(2) cells were plated at a density of 1 × 10^4^ cells per well in 96-well plates. After 24 h, cells were divided two groups and infected with pGC-shANXA2-LV or pGC-vector-LV. 48 h later, cells of two groups were treated with Doxorubicin and Etoposide respectively in the above ten concentration gradients for 72 h. Cell viability was determined using a Cell Counting Kit-8 (CCK-8) reagent (Yeasen, China) according to the manufacturer’s instructions. The absorbance of the samples at a wavelength of 450 nm was measured using a microplate reader (BioTek, USA). Drug-response curve was draw according the result of CCK-8 assay.

### Hoechst33342 staining assay

24-well plates were infused with 4% Poly-L-Lysine (Beyotime Biotechnology, China) solution 4 °C overnight for coating. The next day, NB cells (2×10^4^ per well) were plated 24 h before infection. 48 h after lentivirus infection, treatment of Doxorubicin or Etoposide was performed. 24 h later, cell apoptosis was determined by using the Hoechst 33342 reagent (Yeasen) according to the manufacturer’s protocol. Cell apoptosis was detected under fluorescence microscope (Olympus, Tokyo, Japan) using 348 nm excitation wavelengths.

### Annexin V-FITC/Propidium iodide (PI) for flow Cytometric detection of apoptosis

SK-N-BE (2) cells (1×10^5^ per well) were plated in the 6-well plates, 24 h later, lentivirus (pGC-shANXA2-LV and pGC-vector-LV) infection was performed. 48 h after infection, completed medium containing Etoposide was replaced, and 48 h later, cell apoptosis was detected apoptosis by using the Annexin V-FITC kit (BD, San Diego, CA, USA). According to the manufacturer’s protocol, after cells were detached from the culture plate, they were washed twice with PBS, and were resuspended in 190 μl reaction system of binding buffer. 5 μl of Annexin V and 5 μl of propidium iodide (PI) were added for each sample. Subsequently, the cells were incubated in the dark for 15 min at room temperature. A total of at least 10,000 events were collected and analyzed by flow cytometry (BD FACScanto II) and the apoptotic ratios generated automatically in the Q2 and Q4 quadrant. Due to the fact that the color of doxorubicin was red, which was an interference in the emissional spectrum of PI, we didn’t conduct flow cytometric assay for doxorubicin.

### Extraction of total proteins and nuclear&cytosolic proteins

Total cell proteins were isolated and purified using the Total Protein Kit (Sigma) according to the manufacturer’s protocol. Extraction and isolation of nuclear and cytoplasmic proteins were performed according to the manufacturer’s instructions (Yeasen, China). Briefly, after treatment, cells were washed twice with PBS, scraped and collected by centrifugation at 1500×g for 5 min. Cell pellets were resuspended in 250 μl extraction buffer A and incubated for 10 min on ice. Afterwards, extraction buffer B was added, and samples were vortexed for 30 s at 4 °C. After centrifugation at 12000×g for 5 min at 4 °C, supernatants, which contained the cytosolic fractions, were removed out. The rest pellets, which contained the nuclei, were resuspended in 50 μl of nuclear extraction buffer, and nuclear proteins were extracted by shaking the samples for 30 min at 4 °C. Afterwards, samples were centrifuged at 12000×g for 5 min at 4 °C and the supernatants were removed for analyzing. The protein concentration was determined using the BCA assay method (Pierce, USA).

### Western blotting and quantitative polymerase chain reaction (qPCR) array

Equal amounts of proteins were loaded onto an 10% polyacrylamide gel for electrophoresis and then electro-transferred onto PVDF membranes (Bio-Rad, USA). Blots were blocked with 5% BSA at room temperature for 2 h, and incubated with the appropriate primary antibodies at 4 °C overnight. After washing the blots 3 times, the membranes were incubated with HRP-anti-rabbit IgG for 1 h at room temperature. Proteins were visualized by electrogenerated chemiluminescence (ECL, Pierce Biotechnology, USA) with the Bio-Rad ChemiDoc XRS imaging system. Antibodies were purchased from Cell Signaling Technology (Beverly, USA) and used to direct against ANXA2 (1:1000, #8235), NF-κB1/p50 (1:1000, #13586), Rel-A/p65 (1:1000, #8242), Cleaved-PARP (1:1000, #5625) and AIF (1:1000, #5318), Anti-GADPH (1:2000, #2118) and Histone-H3 (1:2500, #4499) were used as loading control. The results were quantitative analyzed through the software of Image J (X64, v. 2.1.4). Total RNA from cells was isolated with Trizol reagent (Invitrogen, CA, USA). Reverse transcription was performed using the Advantage RT-for-PCR Kit (Takara, Beijing, China). Expression change of three NF-κB target genes (IL-1A, IL-1B, IL-6) in ANXA2-knockdown SK-N-BE(2) cells was assessed by qPCR assay (Takara) according to the manufacturer’s recommendations. These top and bottom primer sequence was provided in Additional file 4: Figure S4.

### Immunofluorescence assay

SK-N-BE (2) cells were seeded in a 24-well chamber slide (3×10^5^ per well). One day later, cells were transfected with lentivirus, and 48 h after transfection, tumor necrosis factor-α (TNF-α, 100 ng/ml) was added for treating 1 h. Then, cells were fixed with 4% paraformaldehyde solution at room temperature for 30 min, and were treated with 0.3% Triton X-100 for 20 min, blocked in 10% bovine serum albumin for 1 h. incubated overnight at 4 °C with primary antibodies NF-κB1/p50 (1:100, #13586, CST) and ANX A2 (1:150, #H00000302-M02, Novus, USA). Cells were washed and incubated in secondary antibody (1:200, Yeasen, China), DAPI (1:5000, Yeasen) was used to label the nuclei. Immunofluorescence was photography and analyzed by the LeicaCTR6000 microscope system combined with Image J (X64, v. 2.1.4) software. The nuclear localization of p50 was quantified according to data from 5 random figures in each group.

### Co-Immunoprecipitation (co-IP)

The pGMLV-CMV-MCS-3*flag-EF1-ZsGreen-T2A-Puro vector (GeneChem, Shanghai, China, Additional file 4: Figure S4e) was used to package the lentivirus, and we used the lentivirus to established the 3*flag-ANXA2 SK-N-BE(2) cell line as well as normal control cell line. Co-IP was performed as the procedure briefly. Lyze the cells with 5 ml pre-cold IP buffer for 30 min and sonicate the lysate 3 min on ice. Then, centrifuge the lysate at 20000 g for 20 min at 4 °C. Collect the supernatant into 15 ml tube and incubate with 50uL mouse IgG beads (Sigma, A0919) at 4 degree for 3hs and then incubate with 50uL Flag M2 beads (Sigma, A2220) overnight at four degree. Wash the beads with 1 ml IP buffer for 8 times. Vortex the beads with 200uL 3*flag peptides (Sigma, F4799) 20 min at RT to elute the target proteins. Acetone to precipitate the proteins overnight (1:8 volume), and then performed Western blotting.

### Animal experiments

Six week-old male nude mice were purchased from Shanghai Slac Laboratory Animal Co. Ltd. Animals were housed in individually ventilated micro-isolator cages and allowed chow and water ad libitum. All experiments were performed after obtaining protocol approval by the Animal Care and Use Committee of Xinhua Hospital, and in compliance with the NIH animal use guidelines. Nude mice were divided into 4 groups of 5 mice each. Each group was injected subcutaneously in the flank with 1 × 10^7^ SK-N-BE(2) cells pre-transduced with either pGC-shANXA2-LV or pGC-vector-LV in 200 μL PBS. Tumor sizes were determined with calipers every week by measuring the length and width. Tumor volumes were calculated according to the following formula: volume (mm^3^) = (length × width2) /2 [16]. When the tumors reached palpable size (about 100mm^3^), there were two groups of mice were started to receive intraperitoneal injection with the doxorubicin (1 mg/kg/every 3 days). The other two groups were received PBS intraperitoneal injection as negative control, the reference for the dose and course of treatment of doxorubicin can be found in Stewart’s and Geng’s work [16, 17]. Eight weeks after Chemotherapy, mice were sacrificed and tumor xenografts were removed, weighed, and fixed in formalin for HE and IHC staining.

### Statistical analysis

Statistical analyses were performed using SPSS version 18.0 software for Windows. The association of ANXA2 expression with clinical features was analyzed by the Pearson χ2 test. Survival analysis was assessed by Kaplan Meier plots and multivariate Cox (Wald test) analysis. One-way ANOVA was used for multiple comparisons. Between-group differences were analyzed using Student’s t test. Values of *P <* 0.05 were considered statistically significant.

## Results

### Global identification of the multidrug resistance-associated proteome

**Fig. 1:**
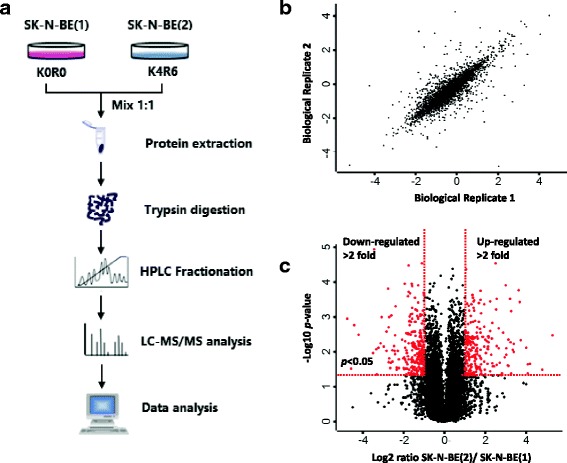
Global identification of a multiple drug resistance associated proteome. **a**. Experimental strategy for global identification of a multiple drug resistance associated proteome. SK-N-BE(1) and SK-N-BE(2) were labeled with K0R0 and K4R6, respectively. Equal number of cells were mixed and lysed. Extracted proteins were digested into tryptic peptides, which were pre-separated and analyzed by HPLC/MS/MS analysis. Proteins were subsequently identified and quantified. **b**. Parson correlation analysis of the quantified proteins between the two replicates. **c**. Volcano plot summarizing protein quantification results in terms of protein changes (log2 fold change > 1 indicates upregulation in the resistant cell line more than two-fold) and significance (*p* value of 0.05 is indicated by the *red line*)

SK-N-BE(1) and SK-N-BE(2) are two stable cell lines derived from the same NB patient but with different drug sensitivities. Proteins that are substantially altered between these two cell lines might directly or indirectly contribute to drug sensitivity. To investigate this possibility, we employed mass spectrometry-based proteomics (Fig 1a). Pearson correlation coefficient of the biological replicates was greater than 0.86, suggesting good reliability of protein quantification (Fig. 1b). In total, 6329 proteins were identified with a false discovery rate of <1% both at the protein and peptide level. Among these, 6230 proteins were quantified (Additional file 5: Table S1). Only proteins up- or down-regulated by greater than two-fold were considered for analysis. This left 460 significantly regulated proteins, including 244 significantly up-regulated and 216 significantly down-regulated proteins (Fig. 1c and Additional file 6: Table S2).

Levels of several known chemoresistance-related proteins were significantly altered (Table 1). MRP-1, the classic chemoresistance related protein, was almost five times more abundant in resistant cells compared with the sensitive cells. Galectin-1 and proteasome maturation protein, both of which are associated with drug resistance in many malignant cancers, showed significant up-regulation in the resistant cell line. Among the down-regulated proteins, we found several candidate proteins associated with drug-resistance, including DNA damage-binding protein 2 and DNA topoisomerase 3-alpha. Based on our datasets, we suggest there are additional proteins related to drug resistance that may be targets of further study.

**Table 1:** Significantly-regulated proteins that are known to associated with chemoresistance in NB cell line SK-N-BE (2) compared with SK-N-BE (1)

Protein ID	Protein name	Gene name	Folds ratio^a^	Related cancer/Tumor
Up-regulated proteins				
P09382 [40, 41]	Galectin-1	LGALS1	12.91	chronic myeloid leukemia, hepatocellular carcinoma, Neuroblastoma
Q9Y244 [42]	Proteasome maturation protein	POMP	6.61	multiple myeloma
P08183 [43]	Multidrug resistance protein 1	ABCB1	4.99	bladder cancer, osteosarcoma, colorectal cancer, breast cancer, neuroblastoma, chronic myeloid leukemia, ovarian cancer, lung cancer, etc.
Q96D09 [44]	G-protein coupled receptor-associated sorting protein 2	GPRASP2	4.68	basal cell carcinoma, medulloblastoma
Q06830 [45]	Peroxiredoxin-1	PRDX1	2.81	pancreatic cancer, cervical cancer
P04049 [46]	RAF proto-oncogene serine/threonine-protein kinase	BRAF	2.72	melanoma, colorectal cancer, gliomas
P49407 [47]	Beta-arrestin-1	ARRB1	2.59	ovarian cancer
P06733 [8, 48]	Alpha-enolase	ENO1	2.40	nasopharyngeal carcinoma, ovarian cancer
P50570 [49]	Dynamin-2	DNM2	2.19	chronic myeloid leukemia
P35080 [50]	Profilin-2	PFN2	2.05	colon cancer
Down-regulated proteins				
Q02880 [51]	DNA topoisomerase 2-beta	TOP2B	0.49	breast cancer, colorectal carcinoma
P28715 [52, 53]	DNA repair protein complementing XP-G cells	ERCC5	0.38	Neuroblastoma, lung cancer, colon cancer, renal cancer, ovarian cancer, etc.
Q13485 [54, 55]	Mothers against decapentaplegic homolog 4	SMAD4	0.33	pancreatic carcinoma, hepatocellular carcinoma, colon cancer, lung cancer
Q92466 [56]	DNA damage-binding protein 2	DDB2	0.21	colon cancer, ovarian cancer
Q13472 [57]	DNA topoisomerase 3-alpha	TOP3A	0.18	ovarian cancer, breast cancer, lung cancer
P34931 [58]	Heat shock 70 kDa protein 1-like	HSPA1L	0.05	osteosarcoma, breast cancer, colorectal adenocarcinoma

Source of Database: Pubmed, ScienceDirect, SpringerLink, SCI-ISI, Ovid-MEDLINE, Wiley-Online-Library, AMA, UniProtKB, InterPro, ProtInc

^a^SK-N-BE(2)/SK-N-BE(1))

### High expression of ANXA2 was associated with greater number of chemotherapy cycles and poor prognosis in NB

Annexin A2 (ANXA2), a multi-compartment protein, is involved in cadherin and S100A10 protein binding, and is known to accelerate plasminogen activation [18]. Recent studies suggest this protein may contribute to drug resistance [19, 20]. In our study, profiling data revealed that ANXA2 was upregulated by more than one order of magnitude in the chemoresistant NB cell line SK-N-BE(2) (Suppl. Table 2). This suggests a potentially important role for ANXA2 in generation of drug resistance in NB. The mechanistic details of that role remain unknown.

**Table 2:** The association between ANXA2 expression with clinical pathologic characteristics in 42 NB patients

		ANXA2 expression			
		Total	Low (*n* = 24)	High (*n* = 18)	*P* value
Gender					1.000
	Male	23	13	10	
	Female	19	11	8	
Age at diagnosis					0.065
	<18 months	23	10	13	
	>18 months	19	14	5	
Primary site					0.109
	Retroperitoneum	34	17	17	
	Postmediastinum	8	7	1	
Stage					0.010
	I-II	20	16	4	
	III-IV	20	7	13	
	IVs	2	1	1	
Lymphatic metastasis					0.012
	Positive	18	6	12	
	Negative	24	18	6	
Histology					0.523
	undifferentiated/poorly differentiated	22	14	8	
	well-differentiated	18	9	9	
	NA	2	1	1	
Bone marrow metastasis					0.000
	Positive	9	0	9	
	Negative	33	24	9	

Low: IHC staining grade 0 ~ 2, High: IHC staining grade 3 ~ 4, *NA* Not Available

**Fig. 2:**
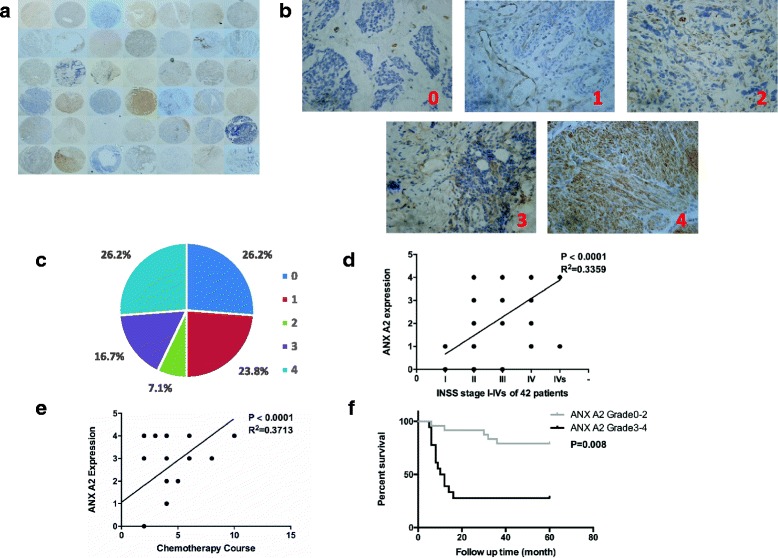
Expression and roles of ANXA2 in NB clinical samples. **a**. A general observation of ANXA2 expression in TMA of 42 NB samples. **b**, **c**. Five grades (0–4) of IHC staining of ANXA2 expression and proportion in NB samples (*n* = 42). **d**. ANXA2 expression is related to INSS stage of NB patients. **e**. ANXA2 expression is related to chemotherapy courses in NB patients. **f**. Kaplan-Meier survival analysis combined with Wald test of NB patients. A high level of ANXA2 was associated with poor prognosis (*n* = 24); a low level of ANXA2 was correlated with a longer survival time (*n* = 18; *P* = 0.008)

To begin investigating the role of ANXA2, we reviewed historical data for each patient in our study. There were 19 girls and 23 boys. Median age at diagnosis was 2.3 years. INSS stage analysis revealed that there were 9 cases with stage I, 11 cases with stage II, 13 cases with stage III, 7 cases with stage IV, and 2 cases with stage IVs. The expression level of ANXA2 in these NB samples was determined by TMA-based IHC analysis. ANXA2 was differentially expressed among those NB samples. In 31 cases (73.8%), ANXA2 protein could be detected. In 42.9% of NB patients we found strong intensity staining (IHC staining grade 3–4, Fig. 2a-c.). We found that strong ANXA2 staining correlated with advanced stage (*p* < 0.0001, r2 = 0.3359, Fig. 2d). We also found that NB patients with higher expression of ANXA2 received more cycles of chemotherapy (*p* < 0.0001, r2 = 0.3713, Fig. 2e). Finally, to assess the prognostic value of ANXA2 expression in NB patients, we generated Kaplan-Meier curves (Fig. 2f). Patients with low expression of ANXA2 had more favorable survival than those with high expression (*p* = 0.008). The association between ANXA2 expression and clinical characteristics are displayed in Table 2. High levels of ANXA2 in NB were markedly associated with some indicators of NB progression, such as lymphatic and bone marrow metastasis.

### ANXA2 contributes to multidrug resistance in NB

**Fig. 3:**
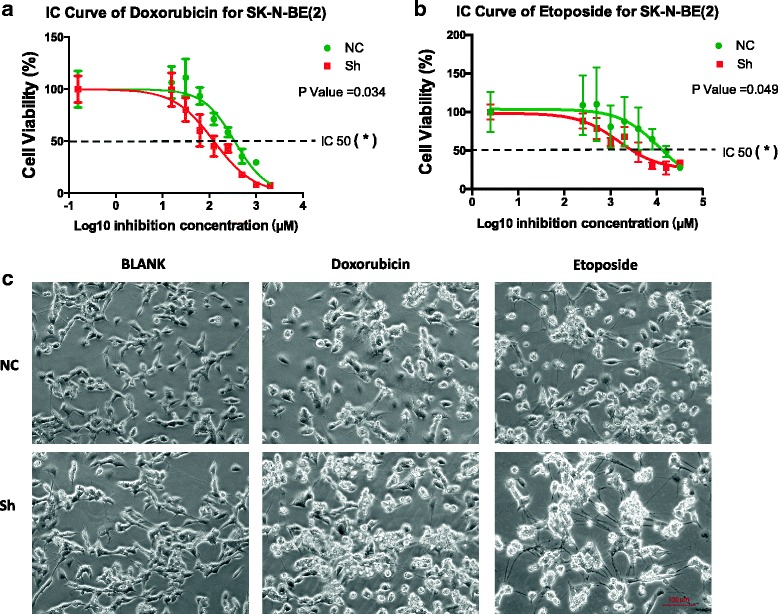
ANXA2 knockdown affected drug sensitivity of NB cells and enhanced cytotoxic effect. **a**, **b**. CCK-8 cell viability assay showed drug response curve was left-shifted in ANXA2 knockdown NB cells. In doxorubicin treatment groups, IC50 value of NB cells was 125.7 nM, 2.77-fold lower than control cells (348.4 nM, *P* = 0.034). Similarly, NB cells with ANXA2 knockdown treated with etoposide had an IC50 value of 1512 nM, which was 7.87-fold lower than the control group (11,894 nM, *P* = 0.049). **c**. Under the microscope, more extensive cytotoxic effects in ANXA2 knockdown cells following treatment with chemotherapy drugs: cell shrinkage, disappearing connections, appearance of small “bubbling” at cell membranes, and apoptotic body formation (Dox, 0.5 μM; etoposide 10 μM for 48 h). *: *p <* 0.05

To investigate the effects of ANXA2 expression on drug sensitivity, we performed a knockdown experiment using shRNA. After comparing reduction efficiency of three candidate shRNA sequences, we choose sequence sh3 for packaging lentivirus (Additional file 2: Figure S2). Using a CCK-8 cell viability assay, we found that the drug response curve was left-shifted in ANXA2 knockdown NB cells, suggesting that these cells were more sensitive to chemotherapeutic drugs (Fig.3a, b). Specifically, the IC50 of doxorubicin in ANXA2 knockdown NB cells was 2.77-fold (*p* = 0.034) lower than that of control cells. Similarly, ANXA2 knockdown NB cells treated with etoposide returned an IC50 value 7.87-fold (*p* = 0.049) lower than that of control cells. On microscopic examination, we found more extensive cytotoxic effects in ANXA2 knockdown cells compared with control cells, following treatment with chemotherapeutic agents (Fig. 3c).

### ANXA2 knockdown induced more apoptosis with chemotherapeutic drugs and activated the apoptotic pathway

**Fig. 4:**
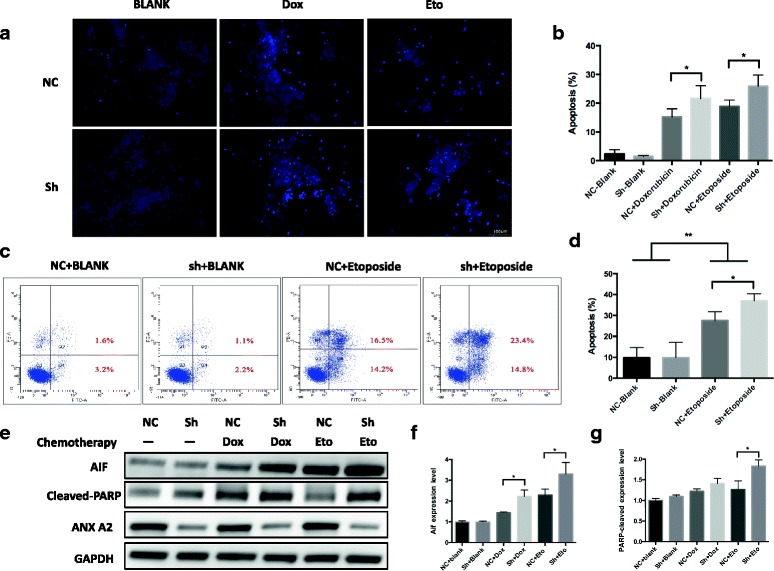
ANXA2 knockdown induced more apoptosis with chemotherapeutic agents and activated the apoptotic pathway. **a**, **b**. After treated with doxorubicin (0.5 μM) or etoposide (10 μM) for 48 h, Hoechest 33342 staining showed significantly increased numbers of apoptotic cells in ANXA2 knockdown group compared with the control group. **c**, **d**. AnnexinV/PI flow cytometric analysis showed significantly increased apoptosis in the ANXA2 knockdown group compared with control group following etoposide treatment. Each quantitative bar chart represents the sum of quadrant 2 and 4 (*n* = 3 independent experiments). **e**~**g**. After treatment with doxorubicin (0.5 μM) and etoposide (10 μM) for 48 h, pro-apoptotic proteins AIF and Cleaved-PARP were increased, particularly in ANXA2 knockdown groups. The plotted error bars represent mean ± SEM from 3 independent experiments. *: *p <* 0.05, **: *p <* 0.01

To investigate whether ANXA2 knockdown had any effect on cell apoptosis following chemotherapy, we performed Hoechest 33,342 staining and flow cytometric analysis. SK-N-BE(2) cells were infected with pGC-shANXA2-LV and pGC-vector-LV prior to treatment with doxorubicin (0.5 μmol/L) or etoposide (10 μmol/L), respectively, for 48 h. As shown in Fig. 4a, ANXA2 knockdown did not induce apoptosis when cells were not exposed to the drugs. However, drug-treated cells transfected with pGC-shANXA2-LV showed greater apoptosis than cells transfected with vector alone (Fig. 4a, b). Annexin V/PI Flow cytometric analysis showed significantly increased apoptosis in the ANXA2 knockdown group compared with control group following etoposide treatment (Fig. 4c, d).

To determine whether ANXA2 knockdown activates an apoptotic pathway, the pro-apoptotic proteins AIF and Cleaved-PARP were measured by Western blot. Following treatment with doxorubicin or etoposide, we demonstrated increased expression of both AIF and Cleaved-PARP in knockdown groups compared with control groups (Fig. 4e~g). These results suggest that ANXA2 may be involved in resistance to chemotherapy-induced apoptosis.

### ANXA2 could regulate the transcriptional activity of the NF-κB signaling pathway through binding to p50 subunit

**Fig. 5:**
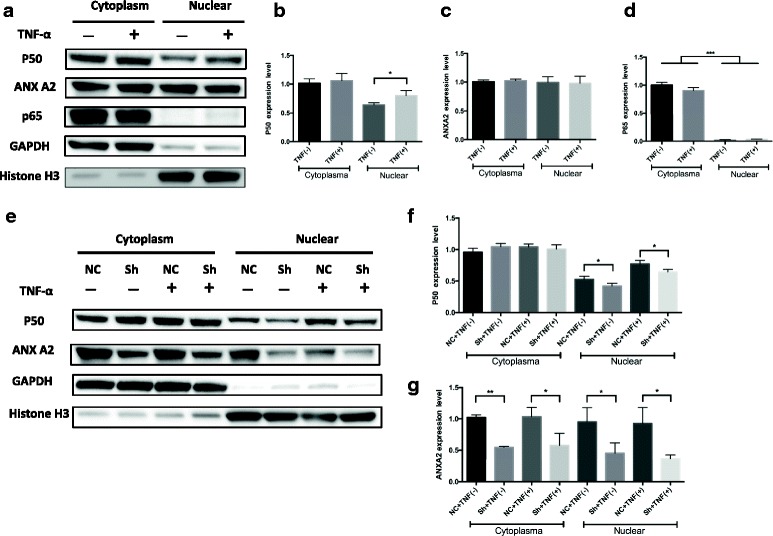
ANXA2 could regulate the nuclear translocation of p50 following treatment with TNF-α. **a**~**d**. The change of subunits (p50, p65) of NF-κB family in SK-N-BE(2) cells treated without or with TNF-α (100 ng/ml) for 1 h. Cytosol and nuclear fractions were prepared special extraction assay. Densitometric analysis showed subunit p50 was involved in NF-κB activation. **e**~**g**. Under the same stimulation of TNF-α, densitometric analysis clearly showed the subunit p50 was downregulated in the nucleus of ANXA2 knockdown NB cells compared with control cells following treatment with TNF-α *: *p <* 0.05, **: *p <* 0.01, ***: *p <* 0.001, The plotted error bars represent mean ± SEM from 3 independent experiments

Recent studies suggested that NF-κB pathway is closely involved in the multidrug resistance of neuroblastoma [21, 22]. We investigated whether ANXA2 regulates drug sensitivity via the NF-κB signaling pathway. First, we performed western blotting to examine which subunits of NF-κB was involved in the activation of NB cells. We found that subunit p50, but not p65, was involved in NF-κB activation (Fig. 5a~d). Further, the results clearly showed the subunit p50 was downregulated in the nucleus of ANXA2 knockdown NB cells compared with control cells following treatment with TNF-α (Fig. 5e~g).

**Fig. 6:**
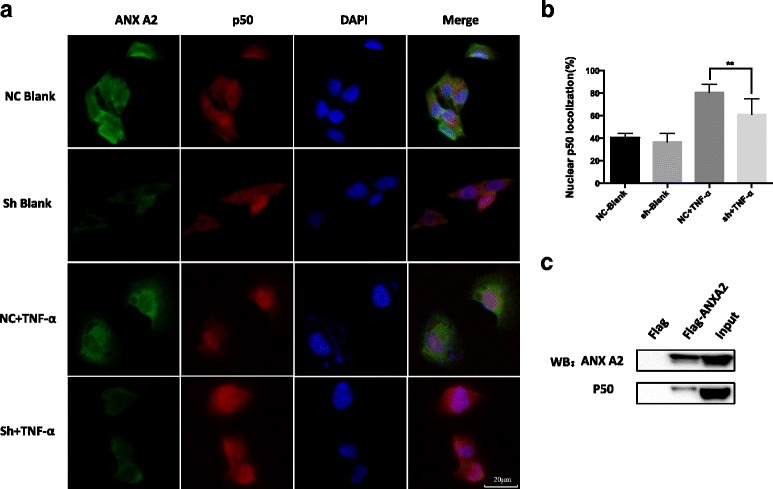
ANXA2 could regulate the activation of NF-κB through combined with the subunit p50. **a**, **b**. The change in localization of endogenous p50 was analyzed by IF-IC following treatment with or without TNF-α (100 ng/ml) for 1 h. Nuclei were stained with DAPI. The nuclear localization of endogenous p50 in each group were quantitated as a graph. The plotted error bars represent mean ± SEM. The nuclear localization of p50 was quantified according to data from 5 random figures in each group. **c**. Interaction between ANXA2 and p50 in SK-N-BE(2) cells was validated through Co-IP and analyzed by western blotting with the indicated antibodies **: *P <* 0.01. All results are from at least three independent experiments

Immunofluorescence staining for subcellular localizations of p50 in resting and stimulating states was examined. In the control group, ANXA2 and p50 were distributed across the cytoplasm and nucleus in the resting state. Following treatment with TNF-α, most p50 translocated to the nucleus. In the ANXA2 knockdown group, the situation was similar in the resting state, however, in response to TNF-α, nuclear translocation of p50 was attenuated (Fig. 6a, b).

To validate the combination of ANXA2 and p50, we performed the co-immunoprecipitation. The results showed clearly that ANXA2 could combine with the p50 subunit directly (Fig. 6c). In addition, in order to consolidate the ANXA2-NF-κB correlation, we selected three well known NF-κB downstream targets (IL-1A, IL-1B, IL-6) for qPCR analysis. Our results showed the IL-1B and IL-6 was decreased significantly in the transcriptional level after ANXA2 knockdown (Additional file 4: Figure S4a~c).

### ANXA2 knockdown could enhance the chemotherapeutic response to doxorubicin in NB xenograft nude mice

**Fig. 7:**
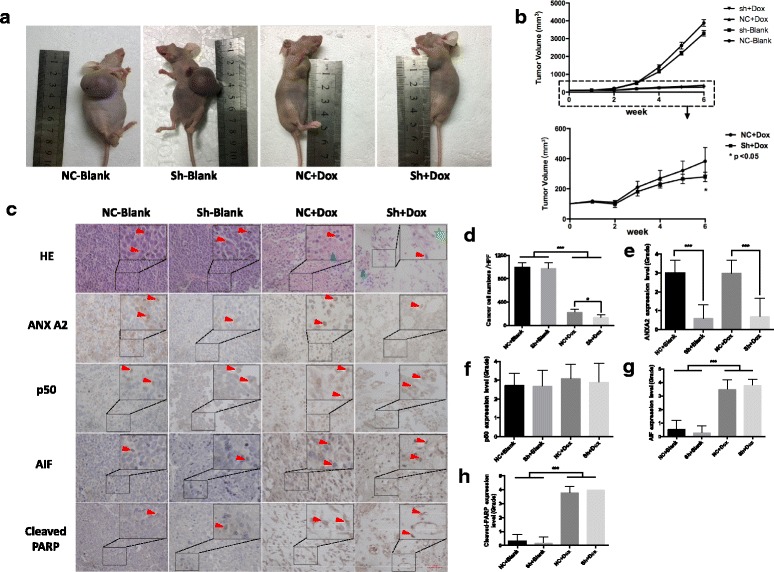
ANXA2 knockdown could enhance the chemotherapeutic response to doxorubicin in NB xenograft nude mice. **a**, **b**. The volume of tumors obtained from animal models were decreased after doxorubicin treatment in vivo, and in ANXA2 knockdown group by shRNA. Efficacy was more substantial compared with control. **c**, **d**. H&E staining of tumor samples of nude mice obviously showed appearance change following chemotherapy: decrease in numbers of small round NB cells (*red arrow*), more foci of hemorrhage and necrosis (*blue arrow*). Particularly in ANXA2 knockdown groups, more cytotoxic effect could be found: less cancer cells and greater degree of vacuolar formation (green pentagram) following chemotherapy. **c**, **e**~**h**. IHC staining showed apoptotic factor AIF and cleaved-PARP proteins were highly expressed in chemotherapy groups. Corresponding quantification of **e**~**h** was performed as previously reported of ANXA2 IHC grade in Fig. 2bc. Original widow is × 400, magnified window is ×800, Bar represents 50 μm, *: *p <* 0.05, **: *p <* 0.01, ***: *p <* 0.001, The plotted error bars represent mean ± SEM, All results are from at least three independent experiments

To further demonstrate the effect of ANXA2 on drug sensitivity in vivo, we generated a subcutaneous xenograft neuroblastoma model. In both control and ANXA2 knockdown groups, tumors were significantly smaller after chemotherapy (Fig. 7a). Further, tumor volume of NB xenografts transduced with Lenti-ANXA2-shRNA was smaller than that of the control (*p* = 0.041). (Fig. 7b). This suggests a positive effect of ANXA2 knockdown on chemotherapy in vivo.

On H&E staining, tumor tissues appeared obviously altered following chemotherapy: decreased numbers of small round NB cells, more foci of hemorrhage and more areas of necrosis. In ANXA2 knockdown groups, NB cells appeared more sensitive to doxorubicin (Fig. 7c~e). Besides that, we measured expression levels of mitochondrial apoptotic pathway components by immunohistochemistry. AIF and cleaved-PARP proteins were highly expressed in chemotherapy groups, but there was no significant difference between control and knockdown with Dox after quantification (Fig. 7c, g~h).

## Discussion

The purpose of this study was to provide new insights into the molecular mechanisms of multidrug resistance in NB. To define the proteome of drug sensitivity-altered NB cells, we utilized SILAC methodology, the current standard for relative quantification of cellular proteomes by MS [23]. In this study, we identified and quantified over 6000 proteins in SK-N-BE(1) and SK-N-BE(2) cell lines, both derived from the same patient prior to and following chemotherapy. To the best of our knowledge, this is the first effort to characterize proteomic profiles of human NB cells with different drug sensitivity. We detected 460 proteins with greatly altered expression. Through bioinformatic analysis, we isolated 16 significantly up- or down-regulated proteins that have been reported to associated with chemoresistance. Our dataset provides a rich source of proteins for identification of drug resistant-related biomarkers, as well as novel targets to overcome drug resistance in NB.

ANXA2 is a pleiotropic calcium and anionic phospholipid-binding protein that exists as a monomer or as a heterotetrameric complex with members of the S100A family. ANXA2 has been proposed to play a key role in many biological processes, including exocytosis, endocytosis, membrane organization and ion channel conductance [24, 25]. Previous studies have suggested that ANXA2 is highly upregulated in many malignant tumors, including breast cancer, renal cell carcinoma, colorectal cancer, hepatocellular carcinoma and pancreatic ductal adenocarcinoma [26–30]. Functional studies suggest that ANXA2 may serve as an independent prognostic marker [31]. Besides that, in could be interact with the other protein and play a critical role in cancer progression, recurrence and metastasis [32].

Recently, Chen et al. [33] found absence of ANXA2 significantly increased chemosensitivity of cisplatin/5-FU/docetaxel/vincristine in nasopharyngeal carcinoma cell lines in vitro. Wang et al. [20] showed that overexpression of ANXA2 in hepatocellular carcinoma cell lines could generate drug resistance to 5-fluorouracil. Although the mechanism is unclear, these above studies suggest that ANXA2 is involved in tumor drug resistance.

In our study, the result of MS showed that ANXA2 expression was 12.52-fold higher in drug-resistant NB cells. To explore the value of this protein in NB about clinical relevance, we performed TMA with 42 cases. TMA results demonstrated that ANXA2 was up-regulated in clinical samples with advanced stage or greater number chemotherapy cycles. High expression of ANXA2 correlated with tumor metastasis and poor prognosis. Our results suggest that ANXA2 could be a prognostic biomarker in NB. The further study revealed that ANXA2 knockdown could decrease IC50 value of doxorubicin and etoposide for several folds in vitro. In knockdown group, the apoptotic pathway is more highly-activated, and we observed a more severe cytotoxic reaction and apoptotic response to chemotherapy. Our results showed suggest that ANXA2 may play an important role in chemoresistance.

To investigate the possible association of ANXA2 to chemoresistance, we performed a global analysis through bioinformatic database and literature review. We assumed that ANXA2 might be involved in drug-resistance through regulation of the NF-κB signaling pathway. Not only because many studies indicated that NF-κB activation is involved in cancer drug-resistance, but also studies have indicated that other ANX family member (ANXA4) could interact with the NF-κB subunit, and ANXA2 has a similar conserved structure domain with ANXA4 [34].

Recently, many researches have suggested NF-κB activation playing an important role in drug-resistance mechanism. For instance: it could help cancer cells escape from chemotherapy-induced cell-death [35]; many downstream transcription factors concerning cycling and apoptosis are disregulated [36]; more stem-like cancer cells are survived with chemotherapy [37]. For chemotherapy resistance in NB, the function of NF-κB is also growing concern recently. Fan et al. reported that treatment with 5Z-7-oxozeaenol could block NF-κB activation and enhance doxorubicin- and VP16-induced apoptosis in NB cells [22]. Wang et al. showed NG25, a synthesized inhibitor of TGFβ-activated kinase-1 (TAK1), induced NF-κB activation in such way as to decrease doxorubicin treatment efficacy in a panel of breast cancer cell lines [38].

Subunit p65 and p50 are the most important subunits of NF-κB, and usually be involved in growth and development of cancer [36]. Due to nuclear translocation of subunits is the key step of activation of NF-κB, we performed nucleus/cytoplasm differential protein extractions, and we firstly showed that in NB cell line SK-N-BE(2), NF-κB could be activated through the p50 subunit following TNF-α treatment, but not p65. Next, we revealed that nuclear translocation of p50 could be attenuated by ANXA2 knockdown in NB cells. Through the co-IP, we confirmed the direct interaction between these two proteins. In addition, qPCR results showed the NF-κB downstream targets IL-1B and IL-6 were decreased significantly in the transcriptional level after ANXA2 knockdown, which indirectly consolidated the ANXA2-NF-kB correlation. To the best of our knowledge, this is the first report suggesting that ANXA2 could regulate NF-κB pathways in NB cells, and it might provide new insight in the correlation between NF-κB and chemoresistance.

It is noteworthy that, the effect of chemosensitivity changing with ANXA2 knockdown seems weaker in vivo: not only minor decrease of tumor volume in knockdown group compared with control group, but also no significance difference with pro-apoptotic factors as AIF and cleaved-PARP. We thought it could be ascribed to three parts as below: Firstly, the method of chemotherapy imitation we used in vivo might be much more cytotoxic. Various drug regimen (dose/frequency/administration) may amplify or decrease the differences among groups. In our study, the dosage of Doxorubicin (1 mg/kg/every 3 days) offered an intense cytotoxic effect to cancer cells. However, it could not perfectly show the effect of ANXA2 knockdown for chemosensitivity changing in vivo. Secondly, the biological process of acquired multi-drug resistance is more complex in vivo than in vitro. More genes as well as signaling pathways could be involved in the process and there might be more complicated in vivo. Thirdly, compared to the patient-derived xenograft (PDX) animal model, our animal model (nude mice with NB cell line-derived tumor) might not so closely simulate the tumor growth and chemotherapy process in clinical. So, In the future studies, different chemotherapeutic regiments with PDX animal models would be attempted and the deeper mechanisms would be studied more.

Recently, Tas et al. reported that chemotherapy-unresponsive patients with gastric cancer had higher serum ANXA2 levels compared with chemotherapy-responsive patients [39]. This suggests that serum ANXA2 level might serve as a diagnostic and predictive marker for response to chemotherapy. Further study is needed to investigate the potential diagnostic and prognostic values of serum ANXA2 levels in NB.

## Conclusion

Our profiling data provided a rich source for further study of the molecular mechanisms of acquired drug resistance in NB, and we screen out the protein ANXA2 could be a prognostic biomarker for NB patients following chemotherapy. ANXA2 can regulate transcriptional activity of NF-κB by binding to the p50 subunit. Knockdown this protein could attenuate multidrug resistance in NB cells through upregulation of apoptotic genes. Taken together, our data suggest that ANXA2 might serve as a potential therapeutic target for NB.

## Additional files

Additional file 1: Figure S1. Morphological observation and IC90 for NB cell line SK-N-BE(1) and SK-N-BE(2) **a.** Morphological characteristics of NB cell lines SK-N-BE(1) and SK-N-BE(2). **b.** IC90 (90% of maximal inhibitory concentration) of multiple current chemotherapeutic drug for these two NB cell lines are significantly different. (PDF 3375 kb)

Additional file 2: Figure S2. Two vector maps for packaging lentivirus and detection of knockdown effect for shRNA-ANXA2 a. The map of vector pGMLV-SC5 RNAi, this vector was used to package lentivirus for isolation of the most effective shRNA among three candidates. **b.** The map of vector pGMLV-SB3 RNAi. This vector was used to package our targeted lentivirus pGC-shANXA2-LV for downstream experiments as it does not possess a eGFP locus, and will not produce interference for flow-cytometry or immunofluorescence assay. **c, d.** Western blotting screened the three shRNAs targeted ANXA2. shRNA3-ANXA2 had the highest knockdown efficiency. (PDF 274 kb)

Additional file 3: Figure S3. The sequences of ANXA2 shRNAs and the difference of IC 50 values between control and shANXA2. **a.** The sequences of three candidate shRNAs target for ANXA2. **b.** A t-test for IC 50 value for Dox (control vs shANXA2) was made and it showed IC 50 values for fitting curves was decreased significantly after knockdown ANXA2. **c.** The similar results for etoposide, IC 50 values for fitting curves was decreased significantly after knockdown ANXA2. (PDF 53 kb)

Additional file 4: Figure S4. qPCR results for NF-kB targets (IL-1A, IL-1B, IL-6) after knockdown the protein ANXA2 and the vector map for 3*flag-ANXA2 in Co-IP. **a.** qPCR results showed the IL-1B and IL-6 was decreased significantly in the transcriptional level after ANXA2 knockdown **b.** The top and bottom primer sequence of IL-1A, IL-1B and IL-6. **c.** The map of vector pGMLV-CMV-MCS-3*flag-EF1- ZsGreen-T2A-Puro vector (GeneChem Shanghai, China). This vector was used to package lentivirus for establishing the 3*flag-ANXA2 SK-N-BE(2) cell line as well as normal control cell line in order to performed Co-IP experiment. (PDF 284 kb)

Additional file 5: Table S1. Protein identification and quantificaiton result of the primary and resistant cell lines by Maxquant. (XLSX 1016 kb)

Additional file 6: Table S2. Significantly regulated protein between priamry and resistant cell lines (*p* < 0.05 and fold of change > 2). (XLSX 98 kb)

## Abbreviations

ANXA2: Annexin A2
CCK-8: Cell Counting Kit-8
COG: Children’s Oncology Group
Co-IP: Co-Immunoprecipitation
ECL: Electrogenerated chemiluminescence
IC50: 50% of maximal inhibitory concentration
IC90: 90% of maximal inhibitory concentration
IHC: Immunohistochemistry
INSS: International neuroblastoma staging system
MRP1: Multidrug resistance protein 1
MS: Mass spectra
NB: Neuroblastoma
PBS: Phosphate-buffered saline
PDX: Patient-derived xenograft; SEM: standard error of the mean
qPCR: Quantitative Polymerase Chain Reaction
shRNA: Short hairpin RNA
SILAC: Stable isotope labeling with amino acids
TAK1: TGFβ-activated kinase-1
TMA: Tissue microarray
