# Supplementary material for: Annexin A2 could enhance multidrug resistance by regulating NF-κB signaling pathway in pediatric neuroblastoma
Source: J Exp Clin Cancer Res. 2017 Aug 16;36:111. doi: 10.1186/s13046-017-0581-6 (PMC5559827; doi:10.1186/s13046-017-0581-6)
Supplement: Supplementary file 1 — Morphological observation and IC90 for NB cell line SK-N-BE(1) and SK-N-BE(2) a. Morphological characteristics of NB cell lines SK-N-BE(1) and SK-N-BE(2). b. IC90 (90% of maximal inhibitory concentration) of multiple current chemotherapeutic drug for these two NB cell lines are significantly different. (PDF 3375 kb) [file 13046_2017_581_MOESM1_ESM.pdf]

Figure S1

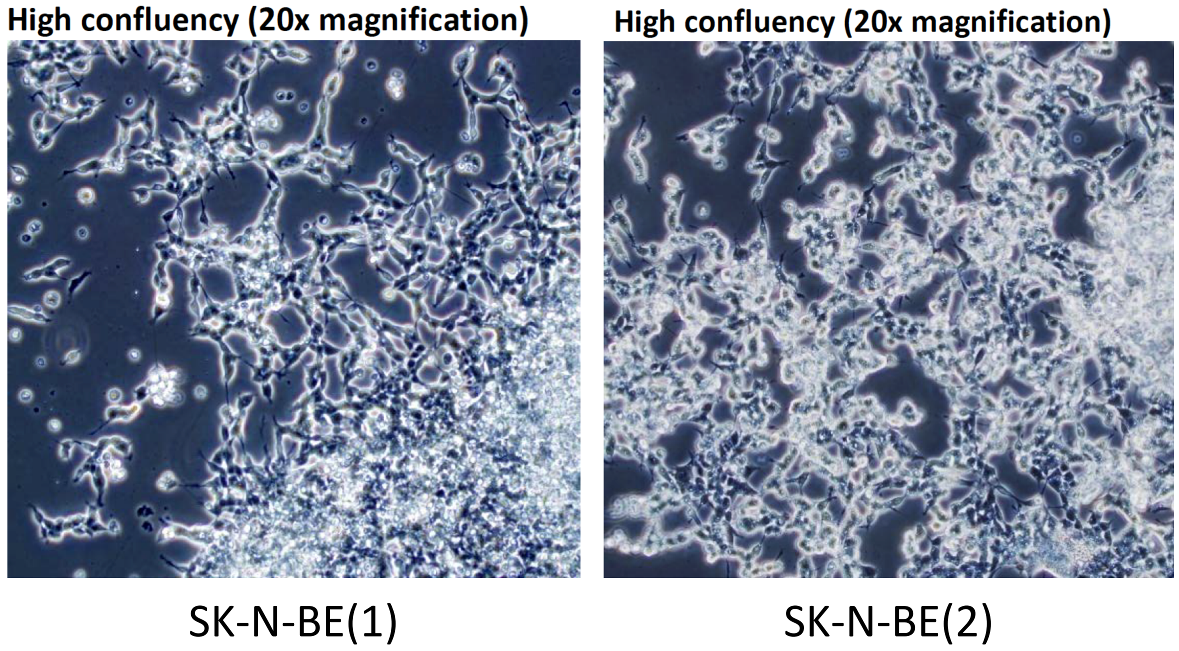

IC90 for SK-N-BE(1) cell line

CBDCA (μg/ml)	CDDP (μg/ml)	DOX (ng/ml)	ETOP (ng/ml)	L-PAM (μg/ml)
0.2	<0.1	<0.1	158	0.8

CBDCA, carboplatin; CDDP, cisplatin; DOX, doxorubicin; ETOP, etoposide; L-PAM, melphalan

IC90 for SK-N-BE(2) cell line

CBDCA (μg/ml)	CDDP (μg/ml)	DOX (ng/ml)	ETOP (ng/ml)	L-PAM (μg/ml)
2.1	0.2	92.3	1130	24

CBDCA, carboplatin; CDDP, cisplatin; DOX, doxorubicin; ETOP, etoposide; L-PAM, melphalan
