# Supplementary figures and images for: Annexin A2 could enhance multidrug resistance by regulating NF-κB signaling pathway in pediatric neuroblastoma
Source: J Exp Clin Cancer Res. 2017 Aug 16;36:111. doi: 10.1186/s13046-017-0581-6 (PMC5559827; doi:10.1186/s13046-017-0581-6)

Figure S2

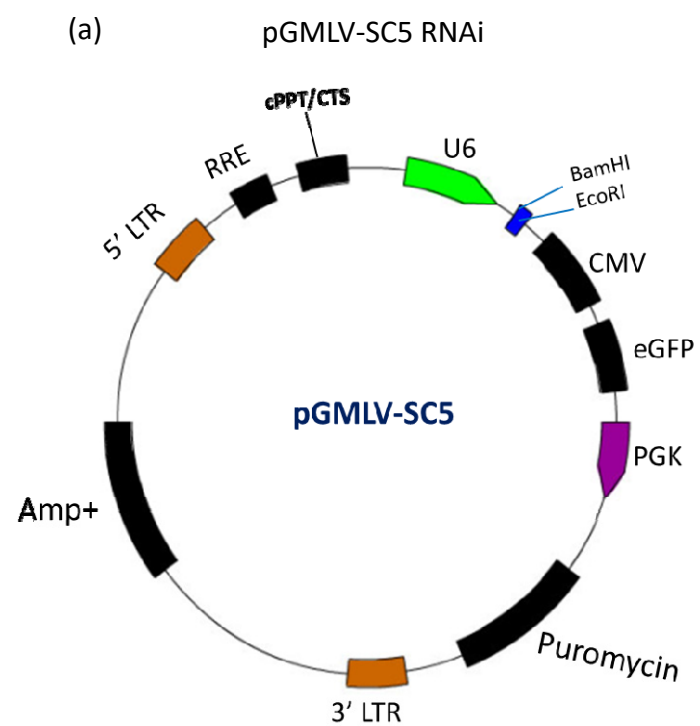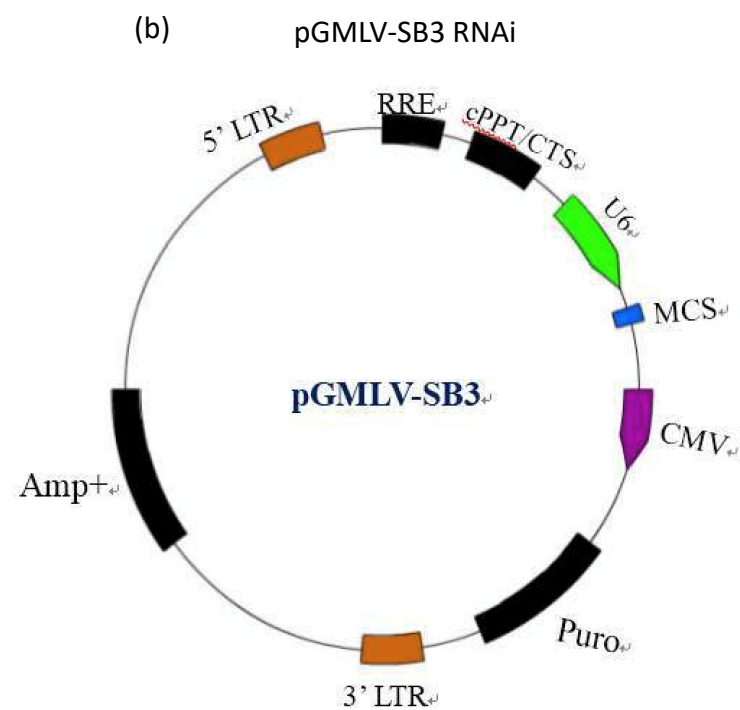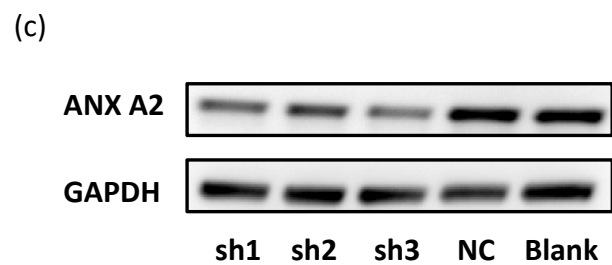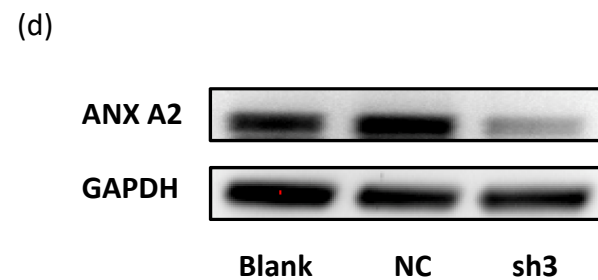

Supplement: Supplementary file 2 — Two vector maps for packaging lentivirus and detection of knockdown effect for shRNA-ANXA2 a. The map of vector pGMLV-SC5 RNAi, this vector was used to package lentivirus for isolation of the most effective shRNA among three candidates. b. The map of vector pGMLV-SB3 RNAi. This vector was used to package our targeted lentivirus pGC-shANXA2-LV for downstream experiments as it does not possess a eGFP locus, and will not produce interference for flow-cytometry or immunofluorescence assay. c, d. Western blotting screened the three shRNAs targeted ANXA2. shRNA3-ANXA2 had the highest knockdown efficiency. (PDF 274 kb) [file 13046_2017_581_MOESM2_ESM.pdf]
