# Supplementary material for: Annexin A2 could enhance multidrug resistance by regulating NF-κB signaling pathway in pediatric neuroblastoma
Source: J Exp Clin Cancer Res. 2017 Aug 16;36:111. doi: 10.1186/s13046-017-0581-6 (PMC5559827; doi:10.1186/s13046-017-0581-6)

Figure S3

(a) sh-1:
Top 5’-GATCCGGAGTGAAGAGGAAAGGAACTTTCAAGAGAAGTTCCTTTCCTCTTCACTCCTTTTTTG’,
Bottom 5’-
AATTCAAAAAAGGAGTGAAGAGGAAAGGAACTTCTCTTGAAAGTTCCTTTCCTCTTCACTCCg’;

sh-2:
Top
5’-GATCCGCATCAGGAAAGAGGTTAAAGTTCAAGAGACTTTAACCTCTTTCCTGATGCTTTTTTG.
Bottom
5’-AATTCAAAAAAGCATCAGGAAAGAGGTTAAAGTCTCTTGAACTTTAACCTCTTTCCTGATGCG-3’;

sh-3:
Top 5’-GATCCGCGGGATGCTTTGAACATTGAACTCGAGTTCAATGTTCAAAGCATCCCGTTTTTT
Bottom
5’-AATTAAAAAACGGGATGCTTTGAACATTGAACTCGAGTTCAATGTTCAAAGCATCCCGCG -3’.

(b)

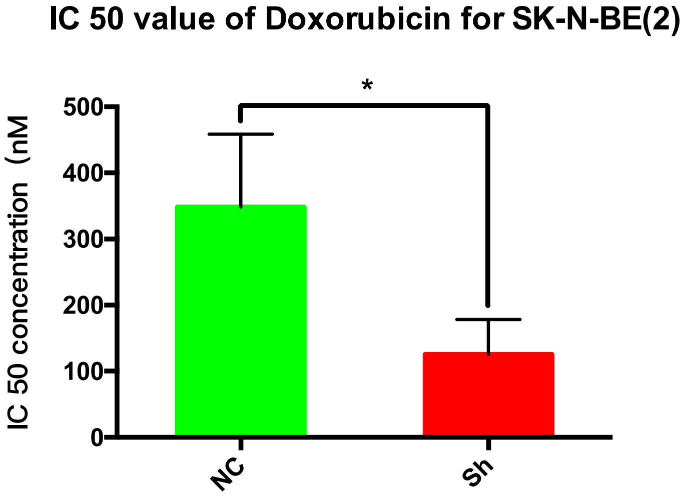

(c)

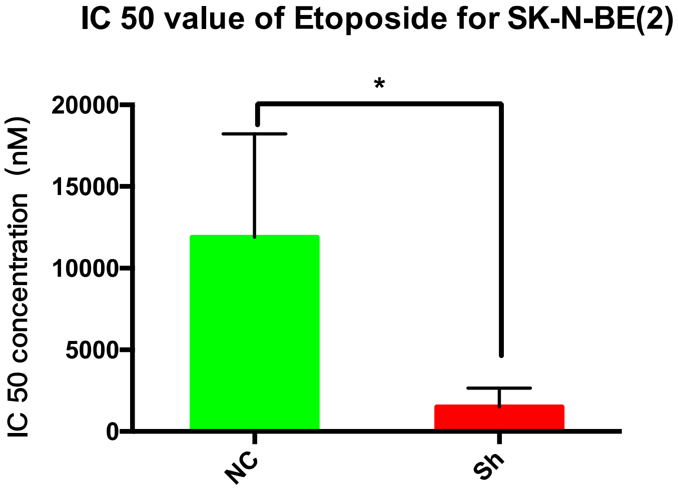

Supplement: Supplementary file 3 — The sequences of ANXA2 shRNAs and the difference of IC 50 values between control and shANXA2. a. The sequences of three candidate shRNAs target for ANXA2. b. A t-test for IC 50 value for Dox (control vs shANXA2) was made and it showed IC 50 values for fitting curves was decreased significantly after knockdown ANXA2. c. The similar results for etoposide, IC 50 values for fitting curves was decreased significantly after knockdown ANXA2. (PDF 53 kb) [file 13046_2017_581_MOESM3_ESM.pdf]
