# Supplementary material for: Annexin A2 could enhance multidrug resistance by regulating NF-κB signaling pathway in pediatric neuroblastoma
Source: J Exp Clin Cancer Res. 2017 Aug 16;36:111. doi: 10.1186/s13046-017-0581-6 (PMC5559827; doi:10.1186/s13046-017-0581-6)

Figure S4

(a)

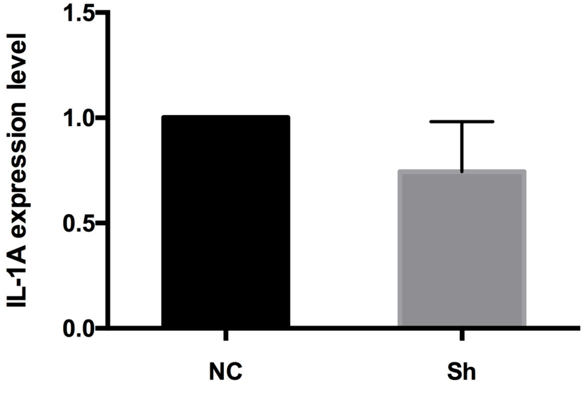

(b)

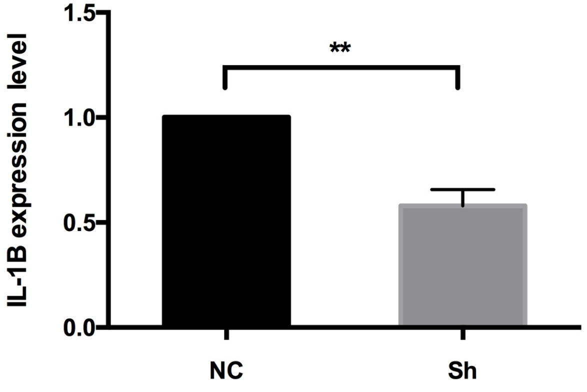

(c)

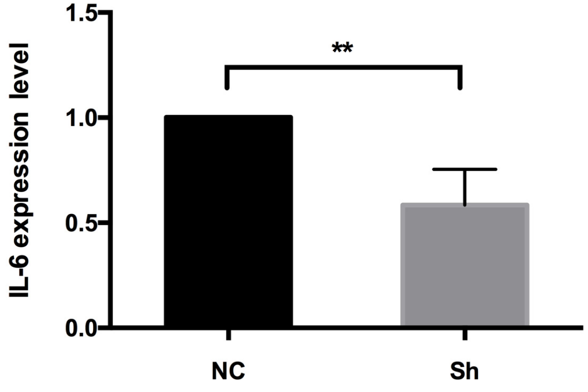

(d)

PCR primer

IL-1A:
Forward: CCTCACCCTCCAACAAAGAT
Reverse: GCCTCAGACATCTCCAGTCC

IL-1B:
Forward: TACAGCAAGGGCTTCAGG
Reverse: TCGTACAGGTGCATCGTG

IL-6:
Forward: CCTCACCCTCCAACAAAGAT
Reverse: GCCTCAGACATCTCCAGTCC

(e)

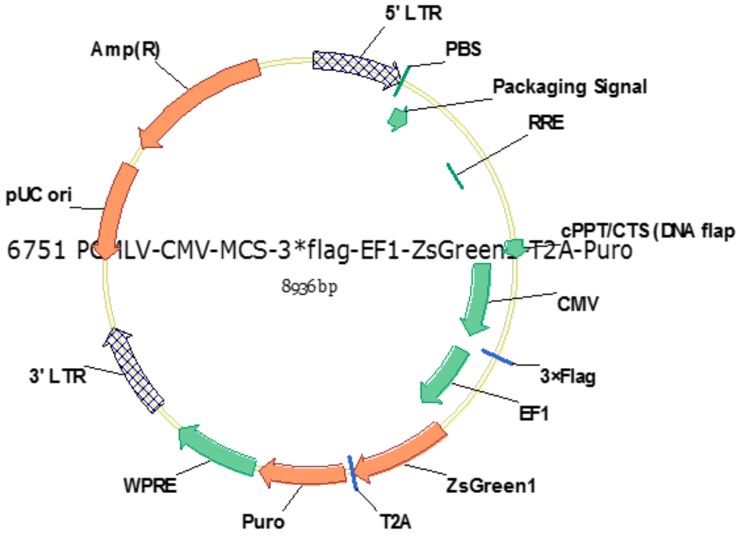

Supplement: Supplementary file 4 — qPCR results for NF-kB targets (IL-1A, IL-1B, IL-6) after knockdown the protein ANXA2 and the vector map for 3*flag-ANXA2 in Co-IP. a. qPCR results showed the IL-1B and IL-6 was decreased significantly in the transcriptional level after ANXA2 knockdown b. The top and bottom primer sequence of IL-1A, IL-1B and IL-6. c. The map of vector pGMLV-CMV-MCS-3*flag-EF1- ZsGreen-T2A-Puro vector (GeneChem Shanghai, China). This vector was used to package lentivirus for establishing the 3*flag-ANXA2 SK-N-BE(2) cell line as well as normal control cell line in order to performed Co-IP experiment. (PDF 284 kb) [file 13046_2017_581_MOESM4_ESM.pdf]
